# Supplementary figures and images for: Targeting NRF2 and FSP1 to Overcome Ferroptosis Resistance in TSC2-Deficient and Cancer Cells
Source: Cancers (Basel). 2025 Aug 21;17(16):2714. doi: 10.3390/cancers17162714 (PMC12384948; doi:10.3390/cancers17162714)

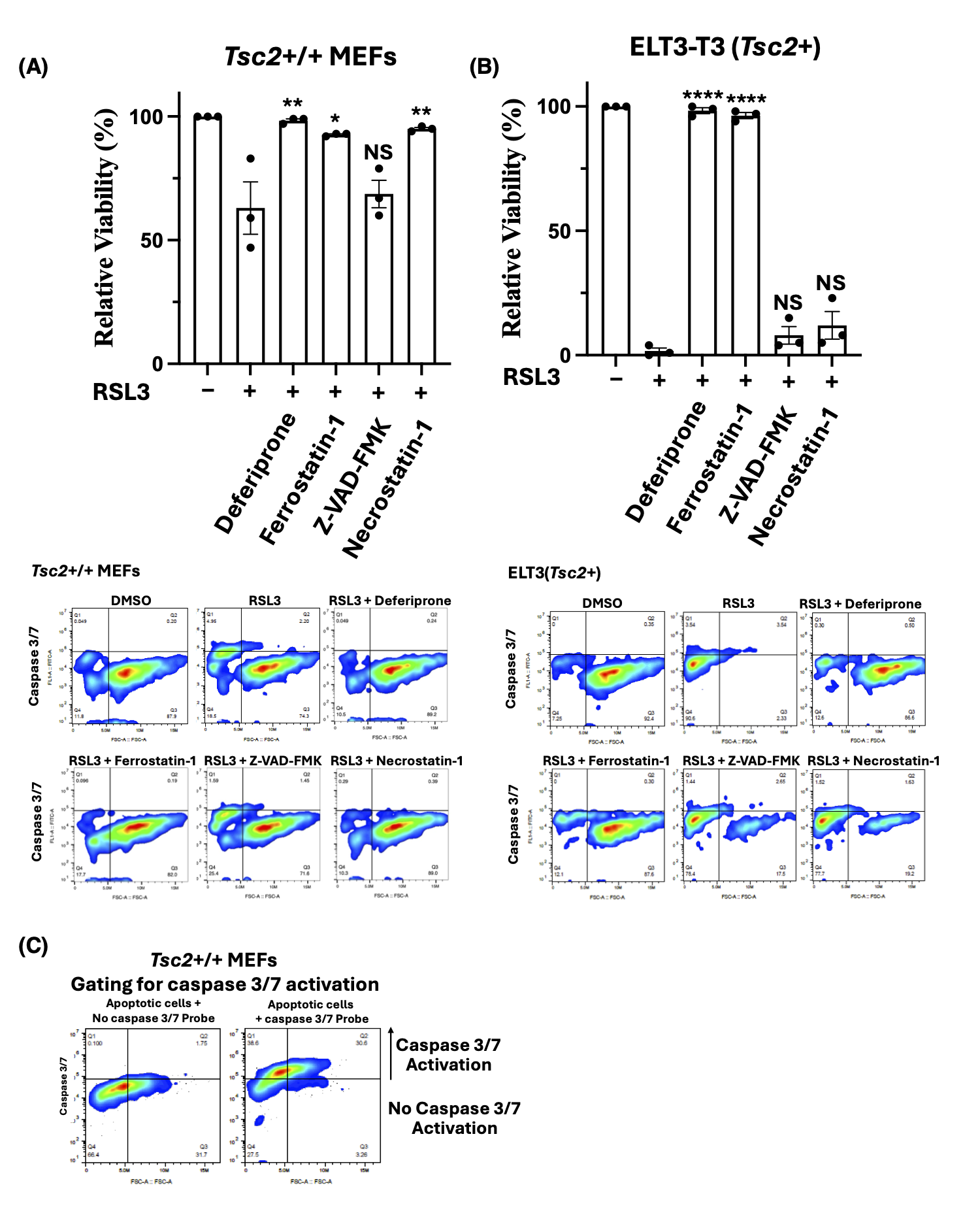

Supplement: Supplementary file 1 [file cancers-17-02714-s001.zip › Figure S1.tiff]

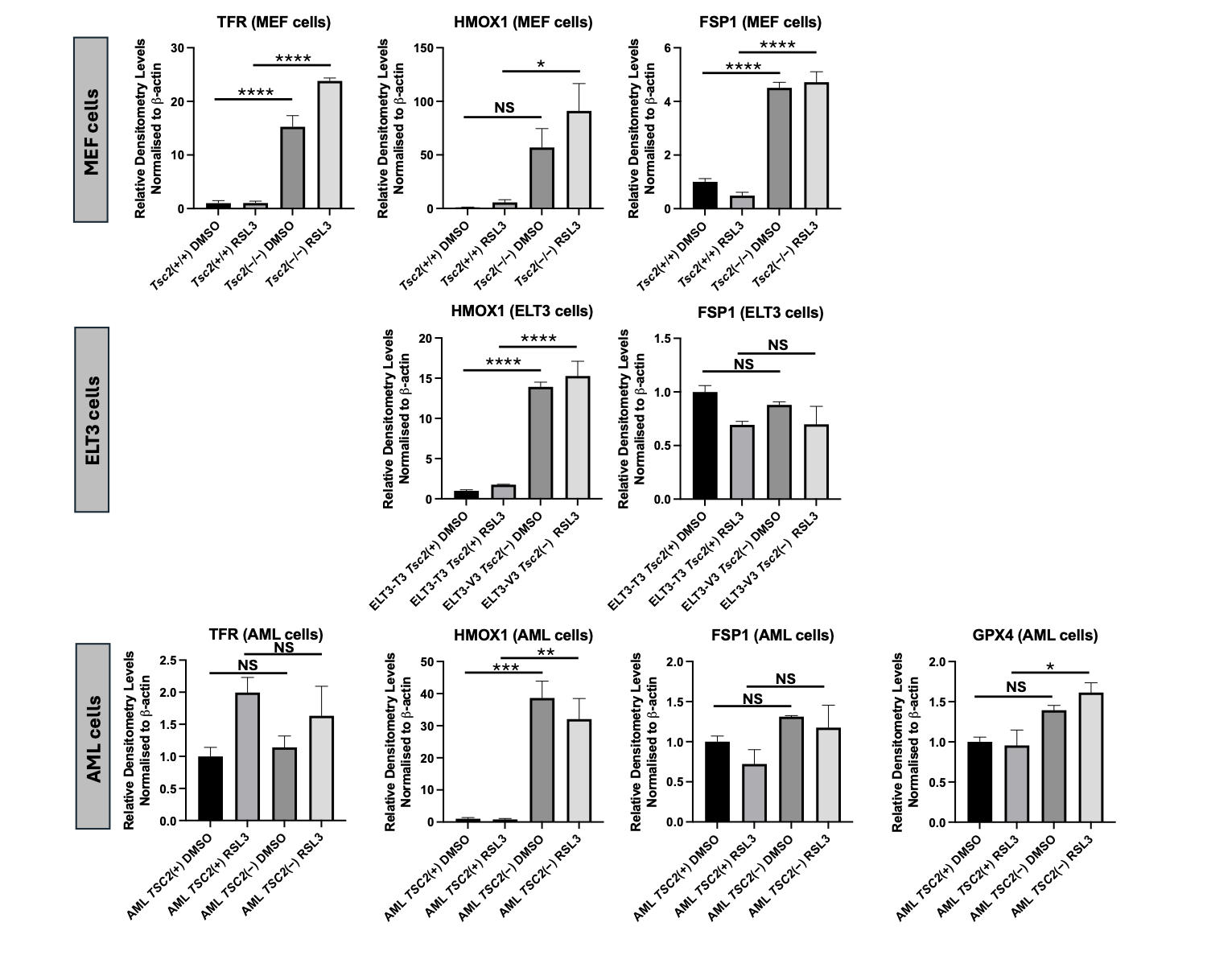

Supplement: Supplementary file 1 [file cancers-17-02714-s001.zip › Figure S2.tiff]

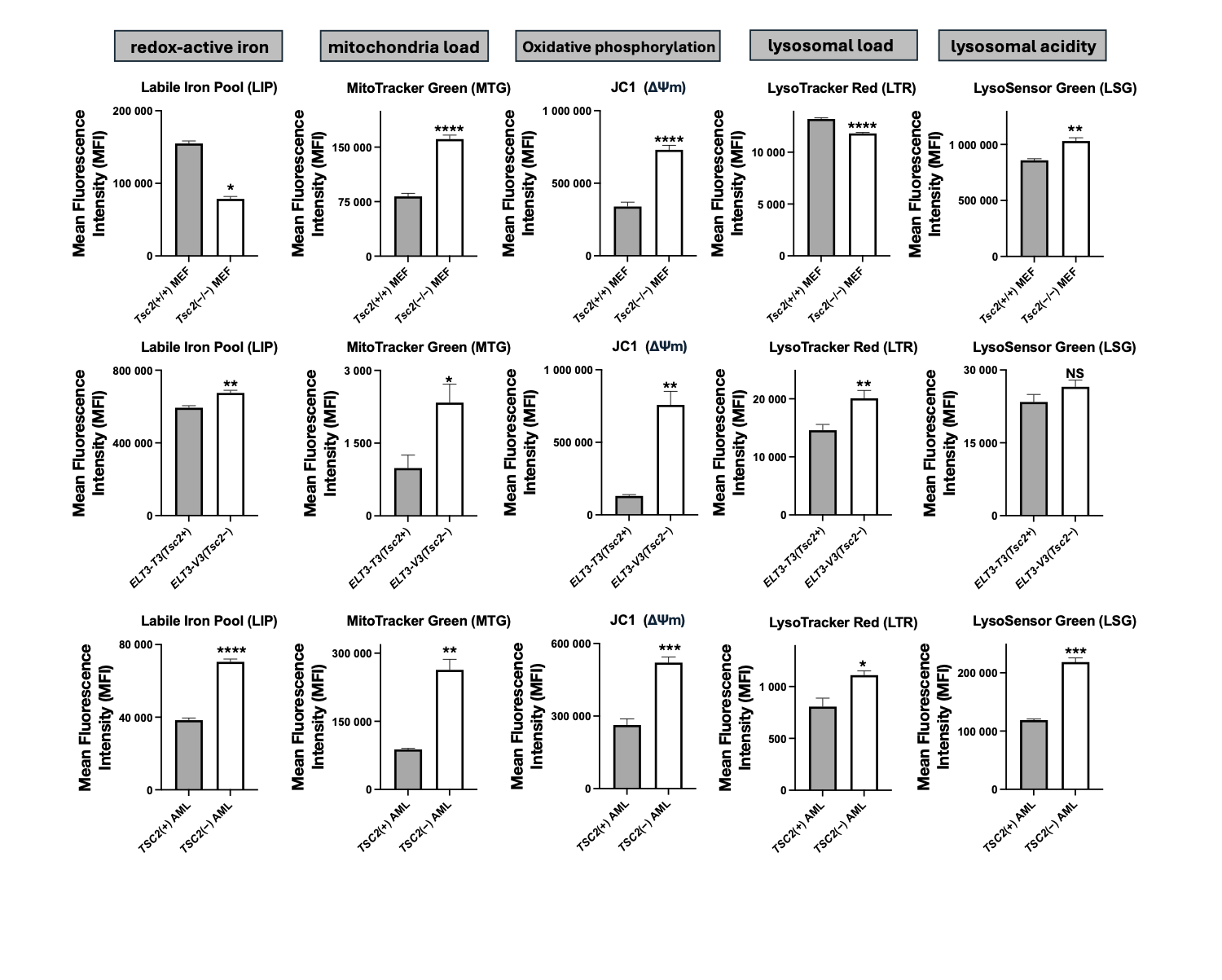

Supplement: Supplementary file 1 [file cancers-17-02714-s001.zip › Figure S3.tiff]

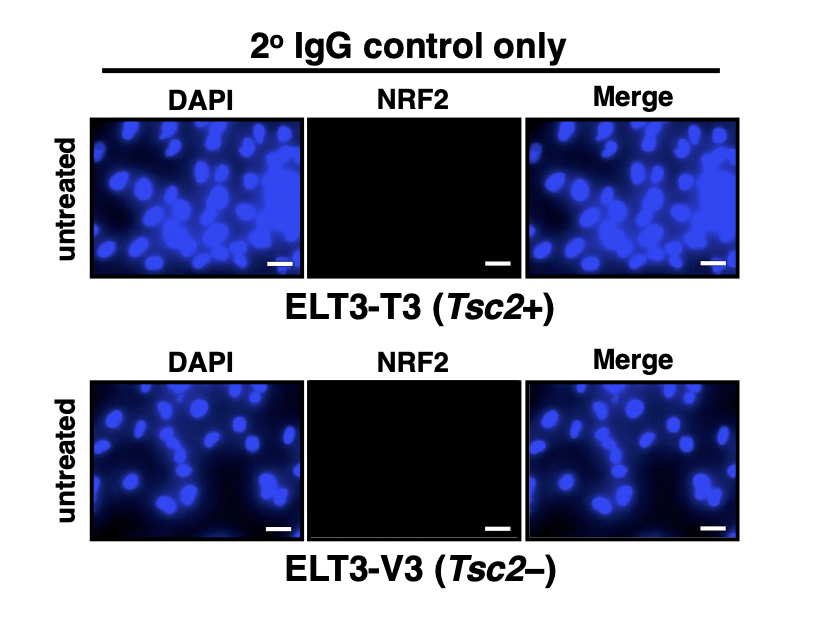

Supplement: Supplementary file 1 [file cancers-17-02714-s001.zip › Figure S4.tiff]

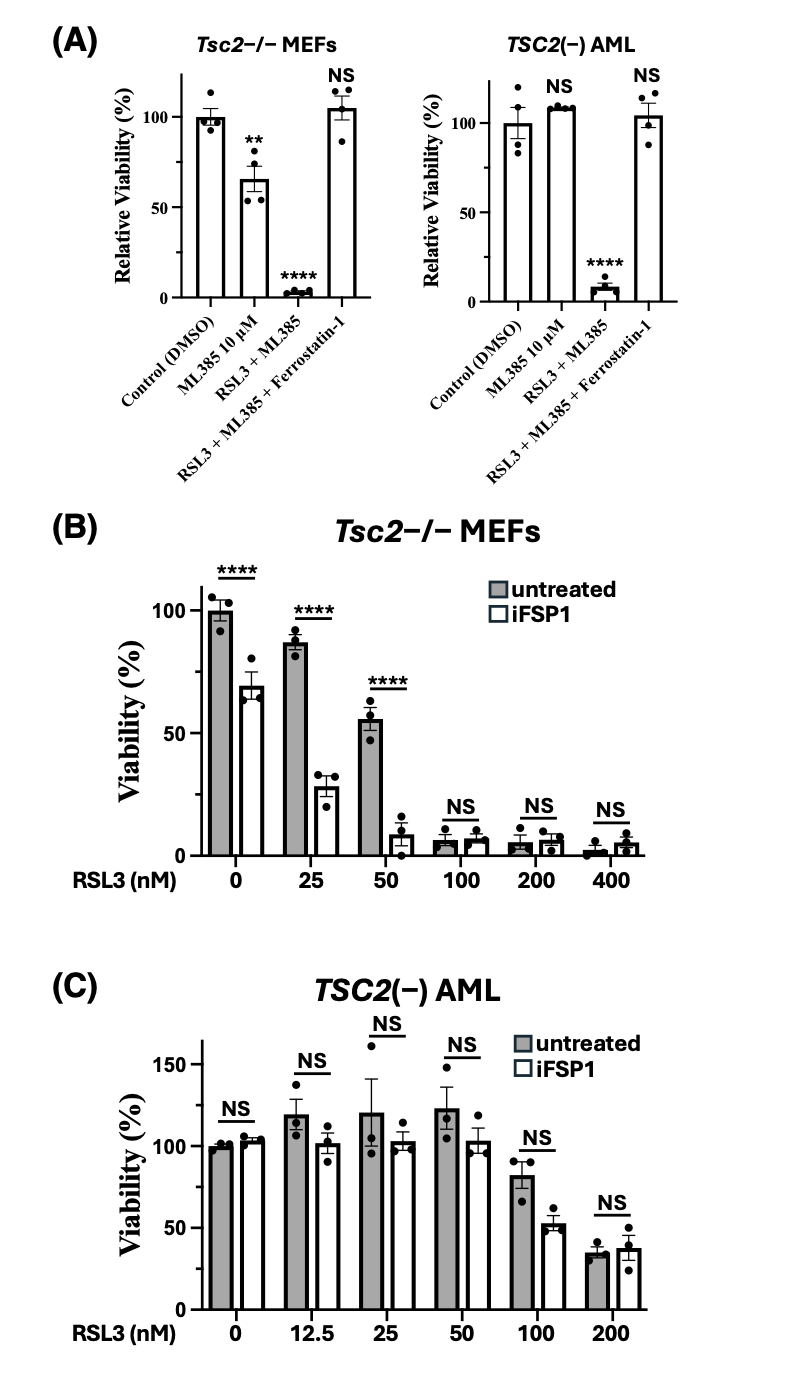

Supplement: Supplementary file 1 [file cancers-17-02714-s001.zip › Figure S5.tiff]

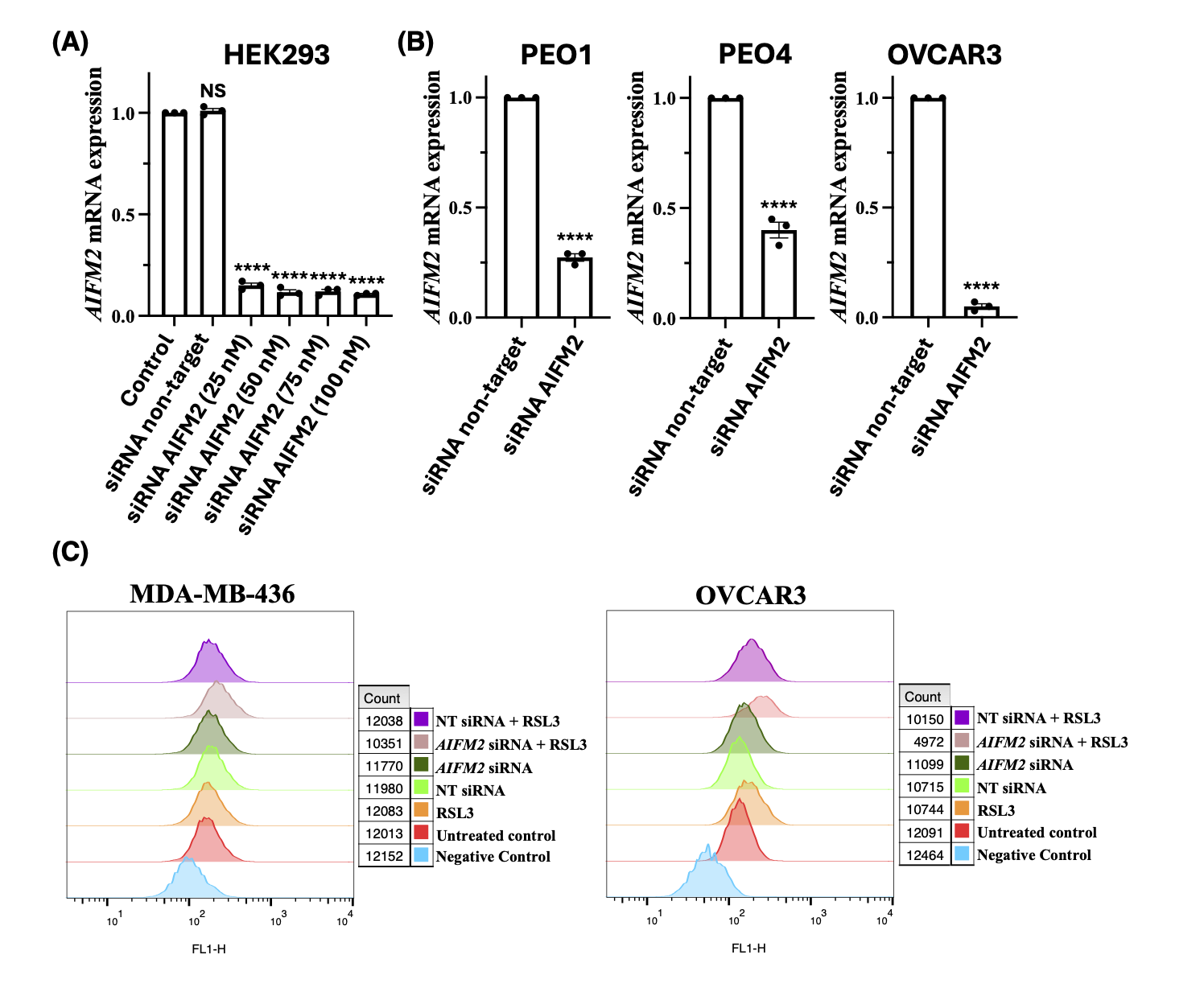

Supplement: Supplementary file 1 [file cancers-17-02714-s001.zip › Figure S6.tiff]

Figure 2C

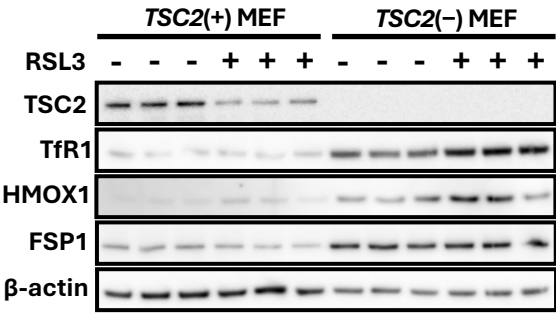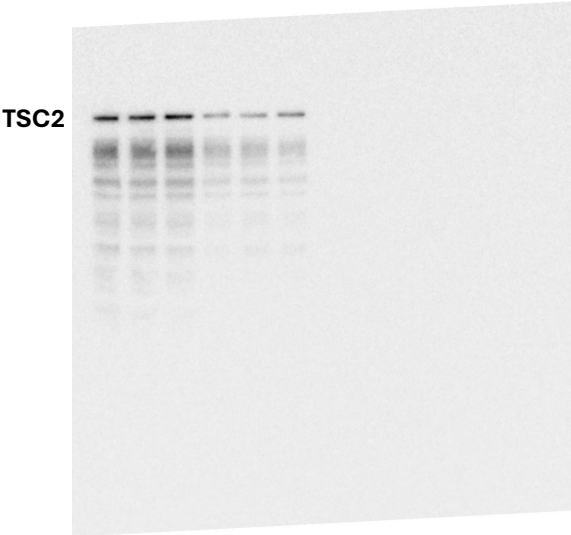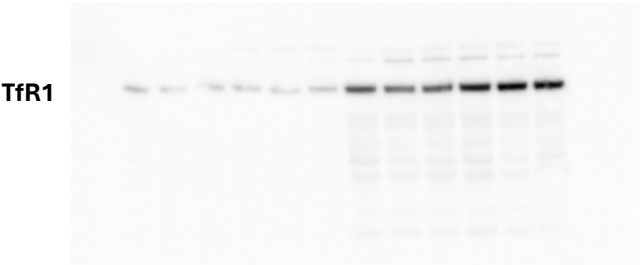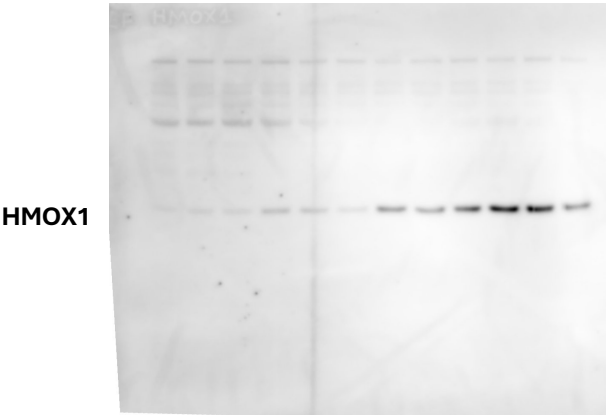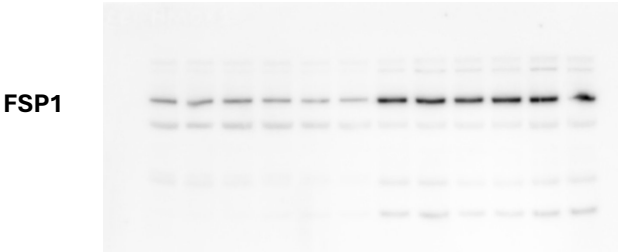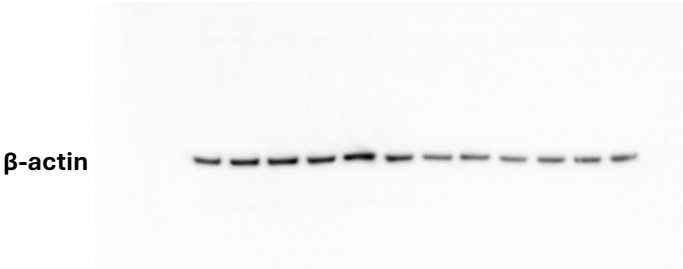

Figure 2D

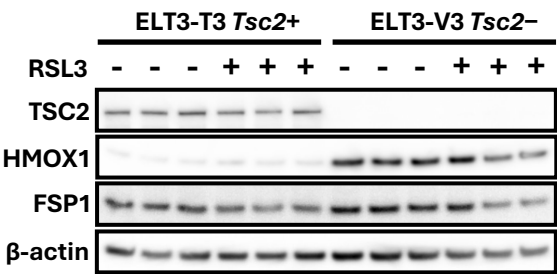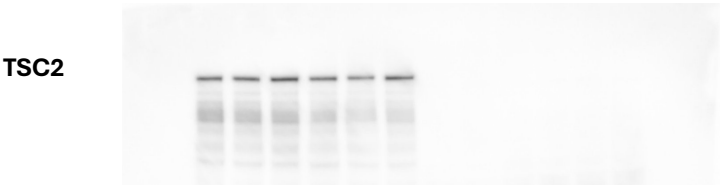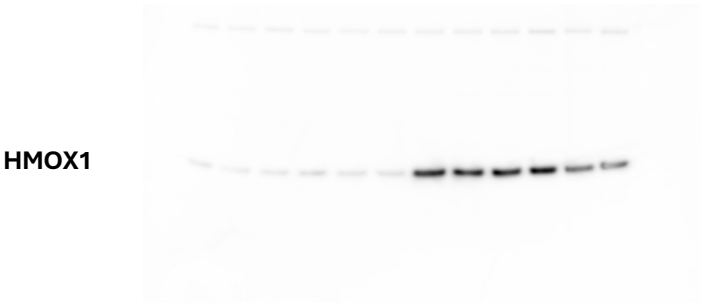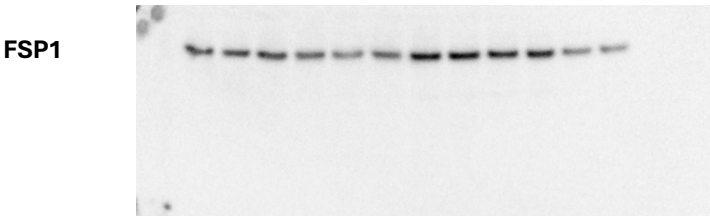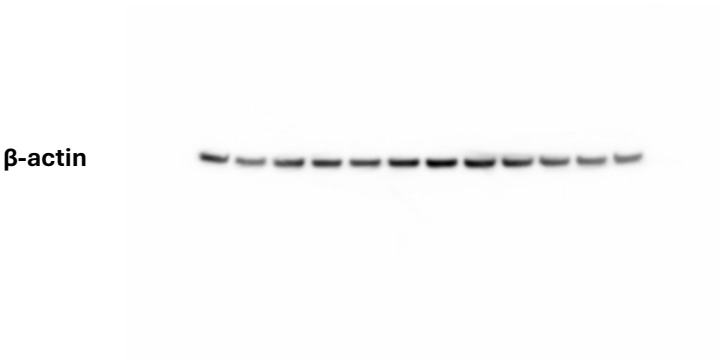

Figure 2E

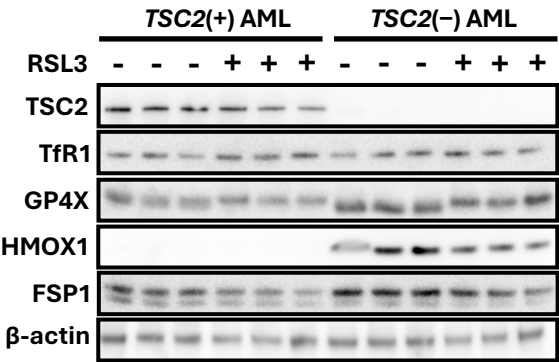

TSC2

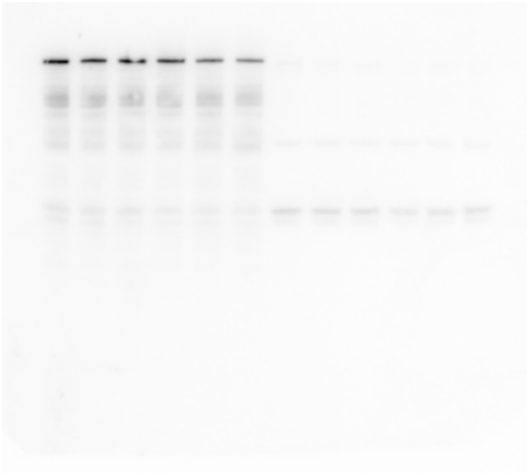

TfR1

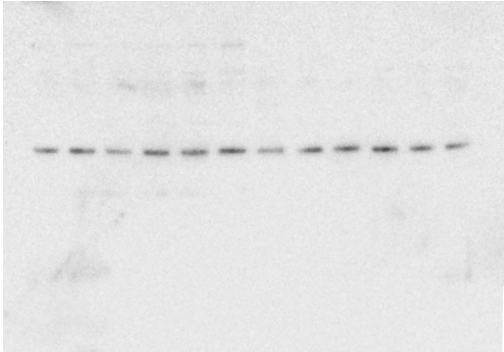

GP4X

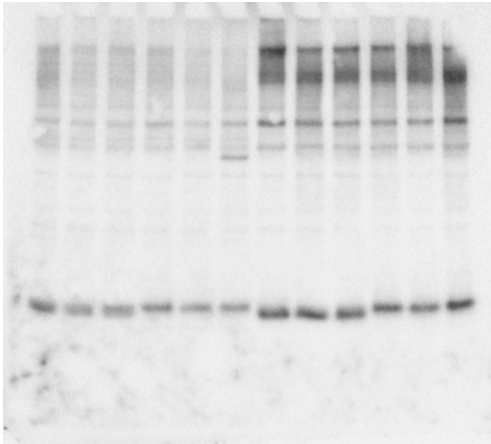

HMOX1

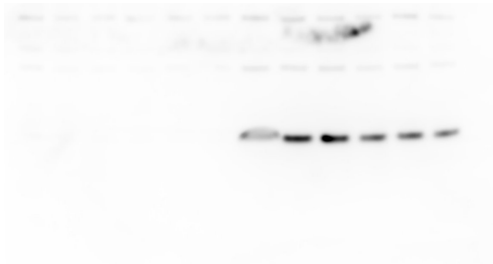

FSP1

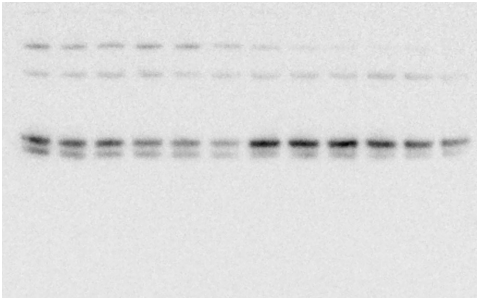

β-actin

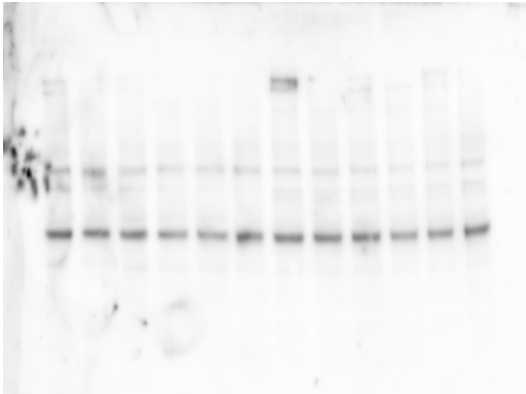

Supplement: Supplementary file 1 [file cancers-17-02714-s001.zip › Supplementary Uncropped Blots.pdf]
